# Preferential Growth of ZnO Micro- and Nanostructure Assemblies on Fs-Laser-Induced Periodic Structures

**DOI:** 10.3390/nano10040731

**Published:** 2020-04-11

**Authors:** Belén Sotillo, Rocio Ariza, Jan Siegel, Javier Solis, Paloma Fernández

**Affiliations:** 1 Materials Physics Department, Faculty of Physics, Complutense University of Madrid, 28040 Madrid, Spain; rocioari@ucm.es (R.A.); arana@ucm.es (P.F.); 2 Laser Processing Group, Instituto de Óptica (IO-CSIC), Consejo Superior de Investigaciones Científicas, Serrano 121, 28006 Madrid, Spain; j.siegel@io.cfmac.csic.es (J.S.); j.solis@io.cfmac.csic.es (J.S.)

**Keywords:** ZnO, fs-laser writing, LIPSS, Raman spectroscopy

## Abstract

In this work, we demonstrate the use of laser-induced periodic surface structures (LIPSS) as templates for the selective growth of ordered micro- and nanostructures of ZnO. Different types of LIPSS were first produced in Si-(100) substrates including ablative low-frequency spatial (LSF) LIPSS, amorphous-crystalline (a–c) LIPSS, and *black silicon* structures. These laser-structured substrates were subsequently used for depositing ZnO using the vapor–solid (VS) method in order to analyze the formation of organized ZnO structures. We used scanning electron microscopy and micro-Raman spectroscopy to assess the morphological and structural characteristics of the ZnO micro/nano-assemblies obtained and to identify the characteristics of the laser-structured substrates inducing the preferential deposition of ZnO. The formation of aligned assemblies of micro- and nanocrystals of ZnO was successfully achieved on LSF-LIPSS and a–c LIPSS. These results point toward a feasible route for generating well aligned assemblies of semiconductor micro- and nanostructures of good quality by the VS method on substrates, where the effect of lattice mismatch is reduced by laser-induced local disorder and likely by a small increase of surface roughness.

## 1. Introduction

Zinc Oxide (ZnO) is a wide bandgap (3.2 eV) II–VI semiconductor material. This semiconductor has the ability to be obtained in the form of micro- and nanostructures with a huge variety of morphologies [1]. Their outstanding luminescent or piezoelectric properties make these ZnO structures one of the main candidates for the fabrication of several micro- and nanodevices [2,3].

Different techniques have been used for the production of micro- and nanostructures [2], using as substrate either the same material [4,5,6] or other substrates like silicon [7,8,9]. In the present work, we chose the thermal evaporation and oxidation of ZnS powders [6] to obtain the micro- and nanostructures of ZnO.

Self-organization of the ZnO structures on the substrate over large areas is desirable for using them in different applications including gas sensing, field emission, and nano-generators, etc. [10,11,12,13]. Different approaches have been proposed for such purposes, the most widely used being the use of a catalyzer [10,14] to obtain ordered structures. A different and also successful approach has been used to obtain self-arranged structures in many other materials. For instance, the patterning by Xe-ion etching has been used to promote the growth of aligned Ag and Au nanoparticle arrays [15,16]. Glass substrates patterned with ultraviolet (UV) nanoimprint lithography have been similarly used to obtain indium tin oxide nanopillar arrays [17]. The use of surfaces covered by laser-induced periodic surface structures (LIPSS) [18] has also successfully been used to produce a heterogeneous and aligned distribution of O/Si ratios along LIPSS structures in length scales of some hundreds of nms by magnetron sputtering at an oblique angle [19].

The generation of LIPSS [20] with short and ultrashort laser pulses has been studied since long ago in a wide variety of materials, from metals to semiconductors and dielectrics [21,22,23,24]. Although the origin of some types of LIPSS is still under debate, there is a general agreement that in the case of low spatial frequency LIPSS (LSF-LIPSS), the phenomenon is associated to interference of the incident laser light with either light scattered by surface roughness or with surface plasmon polaritons (SPPs) [25,26].

In this work, different types of self-organized patterns were obtained on Si substrates upon fs-laser irradiation, depending on the irradiation conditions: amorphous-crystalline (a–c) LIPSS [26]; ablative LFS-LIPSS [25], and *black silicon* [27,28]. All of them have been used as substrates to grow organized arrays of ZnO structures by a simple thermal evaporation method. Successful growth of aligned ZnO micro/nano-assemblies has been achieved on a–c LIPSS and LSF-LIPSS. The morphology and structure of the different laser-processed substrates used, as well as that of the deposited material, were analyzed by SEM techniques and micro-Raman spectroscopy. The results show the feasibility of producing aligned micro/nano assemblies of ZnO structures promoted by laser-induced disorder on the substrate, maintaining the good crystallographic and luminescent properties of the deposited ZnO. The results presented will help in the integration of semiconductors and metals in optoelectronic devices, as will allow the growth of high-quality and organized semiconductor nanostructures on, for example, conducting substrates for electroluminescence devices [29].

## 2. Materials and Methods

**Figure 1:**
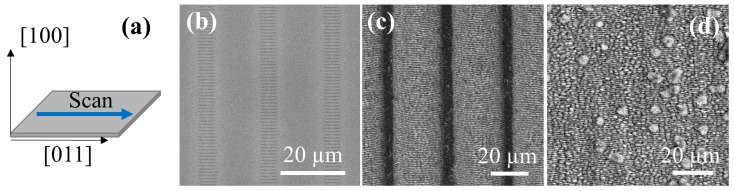
(**a**) Crystallographic orientation of the silicon substrates used. The laser scans were performed along the <011> directions with the polarization parallel to the line scans. (**b**) SEM image of the amorphous-crystalline LIPSS substrate. (**c**) SEM image of the ablative LSF-LIPSS substrate. (**d**) SEM image of the *black silicon* substrate.

Intrinsic silicon (100) was selected to produce the laser-structured substrates. The femtosecond laser used to induce the formation of LIPSS on the surface was an Yb-doped fiber laser operating at a central wavelength of 1030 nm with a pulse duration of 340 fs. The repetition rate was 500 kHz. The experimental setup has been described in detail elsewhere [22,30]. The laser beam goes through a galvanometer beam scanning unit, combined with an F-Theta lens (*f* = 100 mm) for scanning the focused beam over the static sample. The laser spot diameter on the sample was about 48 μm. The silicon samples processed by laser were of 1 cm × 1 cm in size. The laser scans were performed along <011> directions, being the laser polarization parallel to these scan lines (Figure 1a).

Amorphous-crystalline LIPSS [26] (Figure 1b) were formed by scanning the laser over the sample at 2 m/s with a pulse energy of 3.0 μJ (before lens). The separation between scan lines was 20 μm, and the LIPSS period was 1.03 μm. Ablative LSF-LIPSS [25] (Figure 1c) were obtained when the beam was scanned using a pulse energy of 3.4 μJ and a much lower scan speed of 0.2 m/s. The separation between scan lines in this case was set to a larger value (30 μm) to avoid the overlap between consecutive patterned lines. The LIPSS period in this case was 0.84 μm. Finally, *black silicon* [27] (Figure 1d) was produced by scanning the beam at a speed of 0.2 m/s using a pulse energy of 3.6 μJ with a separation between scan lines of only 3 μm and scanning the surface twice. All the parameters used and a comparison of the obtained LIPSS periodicity are listed in Table 1.

**Figure 2:**
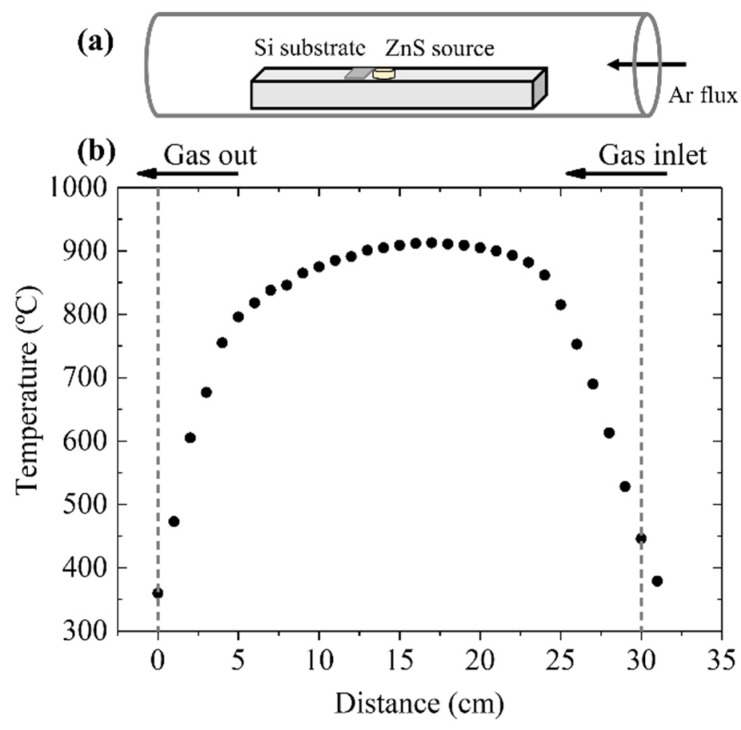
(**a**) Schematic drawing of the furnace tube, indicating the position of the ZnS source, the silicon substrate, and the direction of the Ar flux. (**b**) Temperature profile inside the furnace.

The ZnO structures were grown in a horizontal tubular furnace at a temperature of 900 °C under an argon flux using ZnS powders (Sigma–Aldrich, 99.99% purity, Saint Louis, MO, USA) as precursor. The use of ZnS allowed for performing the thermal treatments at lower temperature [6]. The ZnS powders were compacted under a compressive load to form disk-shaped pellets of approximately 8 mm in diameter. The ZnS source was placed on an alumina boat at the center of the furnace, which was the hottest region, as shown in Figure 2. The temperature was raised from ambient to 900 °C at a rate of 10 °C/min and kept at this value for 10 h. During the thermal treatment, a constant argon flux of 1.5 L/min was maintained. The silicon substrate was placed 6 mm away from the center of the source pellet (located at 15 cm from the edge of the furnace, which was the center position as shown in Figure 2b). The temperature in the furnace was nearly uniform at the position of both the source (ZnS) pellet and the substrate. As the chamber was not evacuated prior to treatment, a certain amount of oxygen remained in the chamber during the growth process, promoting the material oxidation.

The structures obtained were characterized using several SEM-based techniques: secondary electron mode, cathodoluminescence (CL), and electron backscattered diffraction (EBSD). A FEI Inspect S SEM (FEI Company, Eindhoven, Netherlands) or a LEICA 440 SEM (Leica Cambridge Ltd, Cambridge, UK) were used for emissive mode measurements. The EBSD measurements were carried out with a Bruker *e*-Flash Detector in a FEI Inspect SEM working at 20 kV. The analysis of the EBSD data was performed with ESPRIT QUANTAX CrystAlign commercial software (Bruker, Berlin, ESPRIT software version 1.9.4). The Kikuchi patterns, produced by the diffracted backscattered electrons, were indexed by this software to determine the crystal orientation of the sample. The CL measurements were done in the LEICA 440 SEM. For collecting the CL emission, a HAMAMATSU PMA-12 charge coupled device camera (measurement range between 200 and 950 nm), coupled to an optical fiber was used. The CL images were recorded using a R928 photomultiplier attached to a current amplifier Keithley 428 fed into the scanning system of the LEICA SEM. Finally, micro-Raman measurements were carried out in a confocal microscope Horiba Jobin Yvon LABRAM-HR (HORIBA Jobin Yvon, Villeneuve d’Ascq, France) using the 633 nm line of a He-Ne laser. The laser was focused onto the sample using a 100× Olympus objective (0.9 NA), and the scattered light was also collected using the same objective (backscattering configuration). The signal was collected with an air-cooled CCD camera. Raman spectra were collected and analyzed using the Labspec software (France, Labspec version 5.0). All the measurements were done at room temperature.

## 3. Results and Discussion

**Figure 3:**
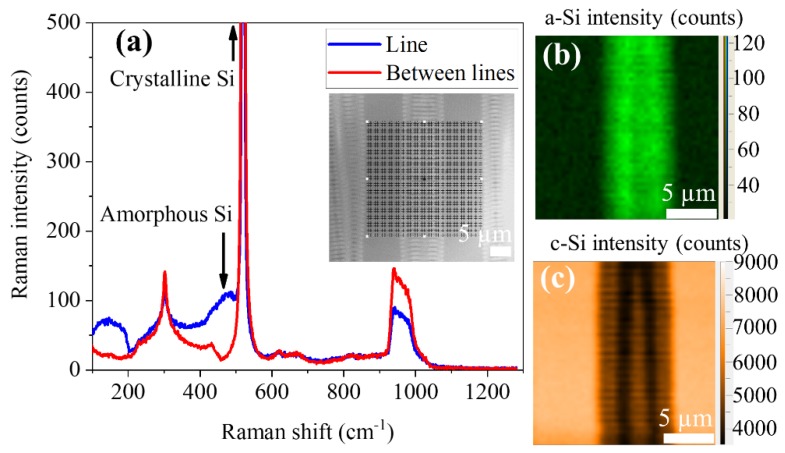
(**a**) Raman spectra recorded on a–c LIPSS substrates. The inset shows an optical image where the grid used for doing the maps in (**b**,**c**) is plotted. (**b**) Map of the amorphous-Si (a-Si) band intensity. (**c**) Map of the crystalline-Si (c-Si) peak intensity.

Prior to their use as substrates for the growth of nanostructures, the irradiated Si samples were characterized by Raman measurements in order to get a deeper understanding of the modifications induced by the laser. The pristine silicon showed the characteristic Raman optical modes [31] centered at 520 cm^−1^ with a width of 4.3 cm^−1^. Second-order bands [31,32] were also detected at 302 cm^−1^ (2TA) and at approximately 965 cm^−1^ (2TO) as can be seen in Figure 3.

In the sample with a–c LIPSS, two different regions were clearly differentiated. In the region between scans, no change in the Raman spectra in respect to those from the pristine silicon (Figure 3a) was observed. On the line scans, where the LIPSS were formed, the intensity of the silicon peak at 520 cm^−1^ was reduced, whereas two new bands appeared centered at 480 and 145 cm^−1^ (see Figure 3a). These two bands were associated to the formation of amorphous silicon [32]. Figure 3b,c show the maps of the region squared in black (see the optical image in the inset of Figure 3a) for the amorphous-Si (480 cm^−1^) and crystalline-Si (520 cm^−1^) peaks’ intensity, respectively. In these maps, the distribution of the two Si phases is clearly appreciated. It can be observed that the crystalline peak intensity decreased in the scan region, whereas the amorphous signal increased in them. The position and width of the crystalline Si peak did not show appreciable changes. A deeper inspection of the scan area indicates that the intensity of both bands had a periodic variation, with a period similar to that observed in the SEM images, thus indicating that the LIPSS were formed by a periodic distribution of crystalline and amorphous silicon, in agreement with the results reported by Fuentes-Edfuf et al. in Reference [33].

**Figure 4:**
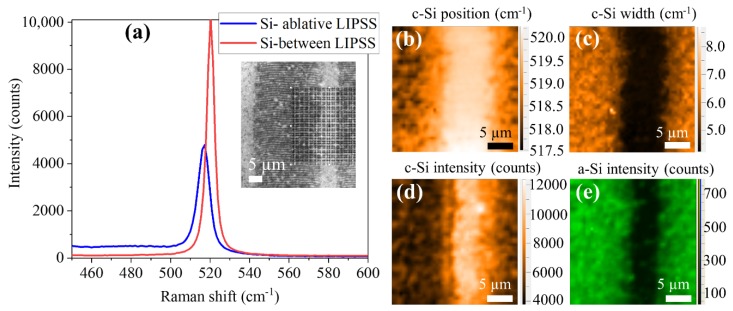
(**a**) Raman spectra recorded on ablative LSF-LIPSS substrates. The inset shows an optical image where the grip used for doing the maps in (**b**–**e**) is plotted. (**b**) Map of the c-Si peak position. (**c**) Map of the c-Si peak width. (**d**) Map of the c-Si peak intensity. (**e**) Map of the a-Si band intensity.

The modifications observed in the ablative LSF-LIPSS samples were much stronger than in the case of the a-c LIPSS. Micro-Raman maps were performed in the area indicated by the grid in the optical image of Figure 4a. Again, we can clearly distinguish the regions ablated by the laser scans from the non-ablated areas between the scans. In the region between scans, the crystalline silicon peak position, intensity, and width are similar those of the pristine material (Figure 4a–d), while closer to the ablated region, bands related to the appearance of amorphous silicon start to appear (Figure 4e). The weak signal of amorphous-Si detected outside the laser scans could be related to material re-deposited from the ablated region. In the region where the ablative LIPSS were formed, the amorphous silicon bands are clearly detected (Figure 4e). Along with a decrease in intensity and an increase of width, a shift of the crystalline silicon peak towards lower wavenumbers is also visible (Figure 4a–d). These features are associated to the presence of tensile stresses in the silicon lattice caused by the violent removal of material leading to the formation of the ablative LIPSS. The shift of the crystalline peak and its increase in width can be ascribed to the formation of polycrystalline silicon [34].

**Figure 5:**
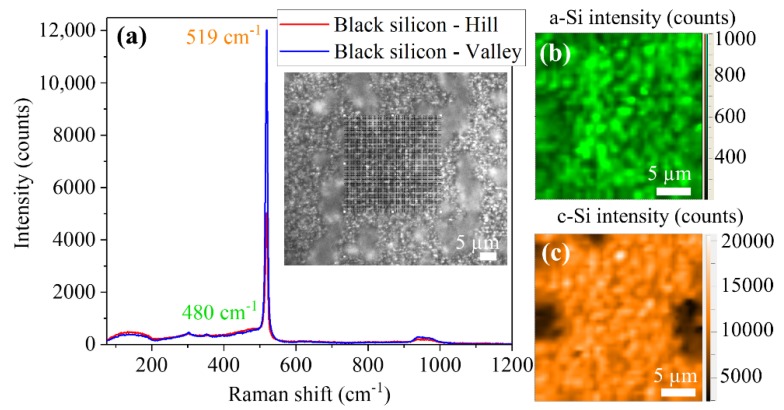
(**a**) Raman spectra recorded on *black silicon* substrates. The inset shows an optical image where the grid used for doing the maps in (**b**,**c**) is drawn. (**b**) Map of the a-Si band intensity. (**c**) Map of the c-Si peak intensity.

Finally, the black silicon samples show an even stronger modification with respect to the initial material. In the irradiated regions, some LIPSS can still be identified, but the entire surface has been modified, in contrast to the case of ablative LIPSS, where stripes of non-modified silicon remain between the laser scan lines. Micro-Raman measurements were performed in the area indicated by the grid in the optical image of Figure 5a to compare the results with the amorphous-crystalline and ablative LIPSS samples. The main effect observed was a shift of the crystalline silicon peak towards lower wavenumbers (519 cm^−1^) all along the irradiated area (Figure 5a). Amorphous silicon was formed as well (Figure 5b). Accumulations of damaged material (hills) were formed over the LIPSS, where the intensity of the Si-peak decreased and its width increased (Figure 5c). Again, the shift of the crystalline peak, along with the increase in its width, and the appearance of the a-Si Raman band is associated with the formation of polycrystalline silicon and the partial amorphization of the material remaining at the surface after ablation.

**Figure 6:**
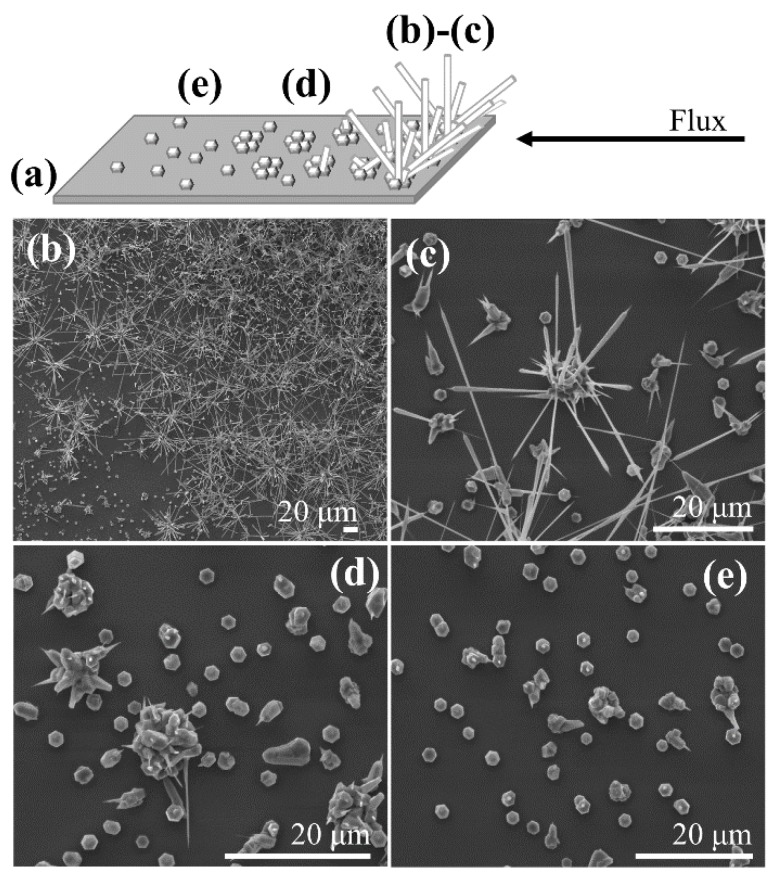
(**a**) Schematic drawing of the distribution of ZnO structures obtained on pristine silicon; (**b**–**e**) indicate the position on this drawing where the corresponding SEM images have been taken.

In order to understand the effect of using laser-patterned silicon substrates, we started our study growing the ZnO structures on a pristine silicon substrate (with the same orientation and properties as the silicon used for laser patterning). In Figure 6, the different types of structures obtained are shown. As a first step, we analyzed the dependence of the morphology of the growth structures on their position with respect to the source (see scheme of Figure 6a). Closer to the source, there was a high density of material deposited (Figure 6b). ZnO was initially deposited forming nucleation clusters, from which the ZnO microrods (with hexagonal cross-section with side lengths in the order of a few microns) grow (Figure 6b). The tips of these rods finish with a nanoneedle (diameter below 100 nm). As the material was deposited farther on the silicon substrate, the rods were shorter or just small needles are observed (Figure 6c). Finally, going even farther from the source, only nucleation clusters are visible (Figure 6d).

From the inspection of the SEM images, the growth process was similar to the one proposed, for example, by Jeong et al. [8]. The growth mechanism was a vapor–solid type, as described below, because of the absence of a catalyst at the substrate surface. In our case, the source material was ZnS which sublimates and dissociates during the thermal treatment. The produced Zn was carried towards the silicon substrate by the Ar flux and deposited randomly on the Si substrate, reacting with the remaining oxygen to form ZnO at the surface. The subsequent arrival of more Zn at the substrate produces the growth of ZnO structures on the pre-existing nucleated clusters as well as the formation of new stable nuclei. The closer to the source, the larger the quantity of material deposited on the nucleation clusters and the longer the ZnO structures grown. The orientation of the structures was determined by the crystalline orientation of the initial nucleation clusters [8], and the growth of the structures on them was promoted by a better lattice matching than with the surrounding Si substrate. Typically, the growth of the ZnO structures is faster along the long direction (*c*-axis, as we show later) than along the lateral directions (i.e., the arriving material is preferentially deposited along the axial direction rather than on the lateral facets of the rod [1]), explaining why the rods typically end in the form of needles.

**Figure 7:**
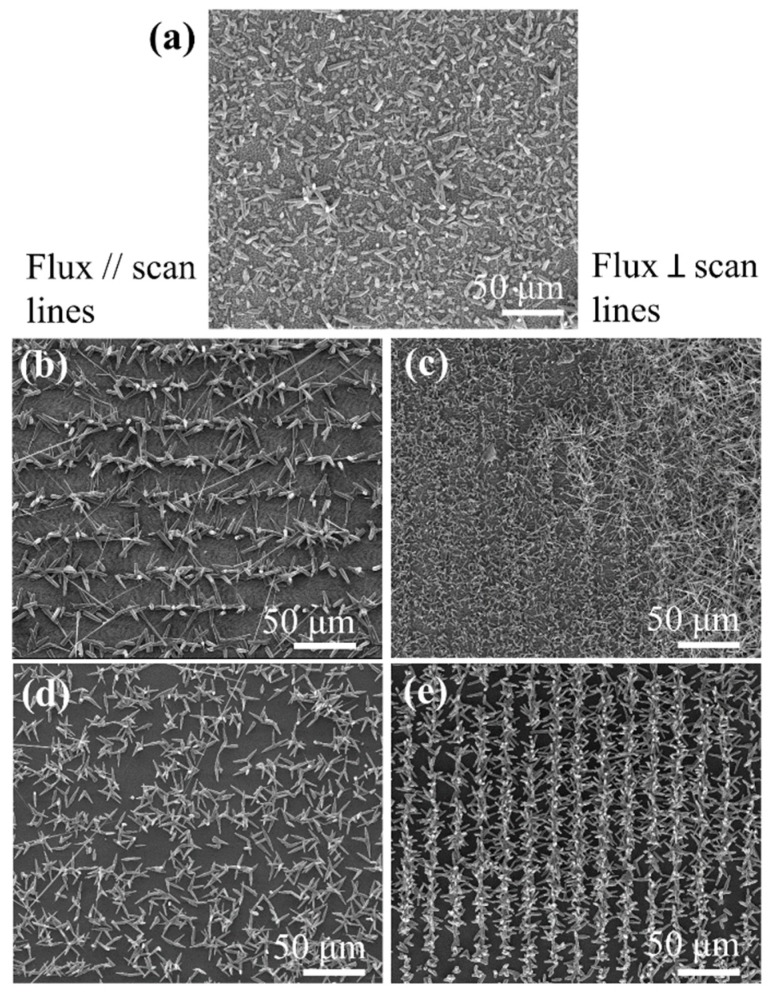
SEM images of the ZnO ensembles of structures grown on the different patterned silicon substrates, showing the effect of the orientation of the scan lines respect to the Ar flux: (**a**) *black silicon* substrates; (**b**,**c**) ablative LSF-LIPSS substrates; and (**d**,**e**) a–c LIPSS. The flux direction is coming from the right of the images. All the images were taken with the same magnification. Microrods have a hexagonal cross-section with a side length in the order of a few microns, whereas nanoneedles have diameters below 100 nm at the tip.

In the case of the laser-structured substrates, the nucleation and growth behavior were conditioned by the laser treatment, i.e., depended on the irradiation conditions and the type of structures induced by the laser. For black-silicon substrates, we obtained microrods at the irradiated area but randomly oriented and distributed (Figure 7a). We observed no difference when placing the substrate with the laser-scanning lines parallel or perpendicular to the Ar flux. In the case of the ablative LSF-LIPSS and a–c LIPSS, the situation was very different, and a clear dependence on the relative orientation between flux and laser scan axis was observed (Figure 7b,c).

On the substrates with ablative LSF-LIPSS, we observed a larger number of nucleation clusters appearing on the non-irradiated stripes between laser scans (Figure 7b). When the scan lines were parallel to the Ar flux, we obtained ZnO microrods growing from the nucleation sites. These microrods typically finished with a sharper tip in the form of a needle. On the other hand, when the scan lines were placed perpendicular to the Ar flux, the structures that grew from the nucleation cluster were more needle like. In this second case, although a kind of preferential growth was observed on the stripes between the LIPSS, random deposition of material was observed on all the regions of the substrate.

Using substrates with a–c LIPSS produced the best results in terms of organization of the nucleation clusters. For both orientations of the sample (scan lines parallel and perpendicular to the flux) the nucleation sites clearly appeared on the irradiated lines (Figure 7c), while nearly no nucleation clusters were formed on the non-irradiated zones. A closer inspection shows that the lines are better defined in the case of the scan lines perpendicular to the flux. For the sample with the lines parallel to the flux, we see that the nucleation was mainly located at the border of the irradiated lines, whereas for the scan lines perpendicular to the flux, the nucleation appears at the center of the irradiated lines. The best organization of the ZnO structures occurs thus for substrates with a–c LIPSS with the Ar flux perpendicular to laser scans.

Taking into account the Raman measurements performed before deposition, we can conclude that ZnO was preferentially deposited on the amorphous Si regions, while deposition on the rough polycrystalline regions associated to ablative-type LSF-LIPSS was less efficient. This can be related to the presence of amorphous silicon, allowing a better matching of the ZnO lattice: the lattice parameters for hexagonal-ZnO were *a =* 3.25 Å and *c =* 5.21 Å, whereas for cubic-Si it was *a =* 5.40 Å. In the case of amorphous silicon, there was not a fixed value for the lattice parameter, as the atoms would be randomly distributed. This will reduce the energy needed for ZnO seeds to be formed on the substrate. Also, it cannot be discarded that the slight increase in the local surface roughness of the a–c LIPSS [26], along with the formation of amorphous silicon, was behind the preferential growth on these regions. This roughness will also reduce the energy needed for the incorporation of ZnO on the substrate. This interpretation is further supported by experiments performed using a Si substrate with different orientation. The ZnO structures were grown onto a–c LIPSS on Si-(111) obtaining similar results as on Si-(100). The orientation of the silicon substrate crystal did not play a critical role on the organization of the ZnO nucleation clusters on the treated surface. This observation indicates a route for orienting the ZnO structures based on reducing the lattice mismatch with the substrate by laser-induced disorder and slight increase in local surface roughness on the substrate.

**Figure 8:**
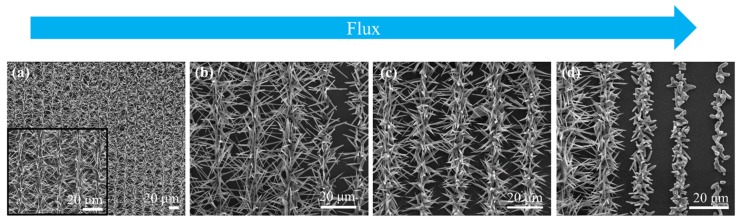
SEM images showing the evolution of the ZnO structures’ shape, grown on the a–c LIPSS substrate, along the direction of the Ar flux (blue arrow). On the left (**a**): the obtained structures closer to the source and gas inlet; in the middle (**b**,**c**): structures obtained in intermediate regions; and on the right (**d**): structures appearing far away from the source.

For all substrates, we observed a strong effect of the distance between the source and the position on the silicon substrate where the ZnO was deposited. This effect was more pronounced in the case of the amorphous-crystalline LIPSS substrate with the flux entering perpendicular to the scan (Figure 8). Closer to the source (Figure 8a), there was a very large amount of material deposited. Despite the high density of structures formed, the nucleation clusters were always localized on the amorphous-crystalline irradiated lines, hence the arrays of ZnO structures were organized in parallel lines. The structures had a needle-like shape, easily seen by moving farther away from the source, where the density of structures formed is reduced (Figure 8b). Looking at even farther regions of deposition (Figure 8c), a slight change in the shape of the structures is observed: in Figure 8c, it can be seen that the structures have a rod-like shape at the base and a needle-like shape at the tip. Finally, far enough from the source, the structures evolve towards a rod-like shape (Figure 8d), and they reduce their length until only nucleation clusters are visible, similar to the case of the pristine silicon substrate but with the clusters organized only at the irradiated lines.

**Figure 9:**
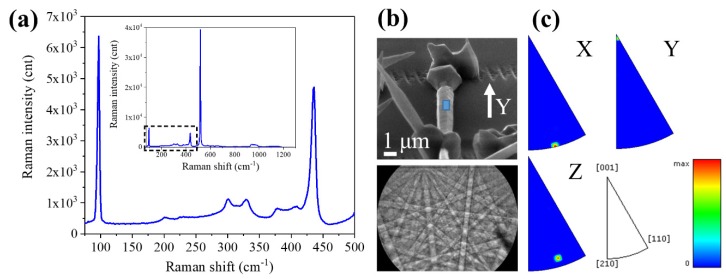
(**a**) Typical Raman spectra of the ZnO structures, recorded using a 632.8 nm laser and a 100× objective, in a backscattering configuration. (**b**) EBSD measurements performed on a rod. SEM image of a selected rod (top left), typical Kikuchi pattern (bottom left), and inverse pole figures (IPFs) for the three axes: *x*, *y*, and *z* (**c**). The IPFs show that the <0001> crystallographic direction was oriented along the *y-*axis.

To get a deeper insight into the nucleation and growth processes, we also characterized the crystallinity and luminescence properties of the ZnO structures obtained. Firstly, we performed micro-Raman spectroscopy along the ZnO structures. All the structures, irrespective the kind of substrate used, the distance from the source or their specific morphology, showed very similar Raman spectra, as the one shown in Figure 9a. As we used a 632.8 nm laser, well below the bandgap of ZnO, the Raman signal from the silicon substrate was still detected. By comparing with the spectra of the Si substrates in Figure 3, Figure 4 and Figure 5, we can ascribe the peaks at 302, 520, and 965 cm^−1^ to the substrate. Selecting the wavenumber region between 75 and 500 cm^−1^, we observe the most important peaks related to ZnO (see Figure 9a). The assignments were the following: 98 cm^−1^ was the E_2_^low^ mode, associated to vibrations of the oxygen sublattice and 438 cm^−1^ was the E_2_^high^ mode, related to the vibrations of the Zn sublattice. At 379 and 409 cm^−1^ were localized the polar modes A_1_(TO) and E_1_(TO), respectively. Peaks at 203 and 331 cm^−1^ were associated to multiphonon processes [35]. The appearance and position of these peaks confirm that the grown structures were wurtzite-ZnO with high crystal quality [35,36], no matter the laser surface treatment performed.

Further crystallographic characterization was performed using EBSD. In order to perform a correct identification of the crystal orientation, the selected ZnO structure was oriented with its long axis along the *x*- or *y*-axis of the reference system of the microscope. As an example, in Figure 9b we show the SEM image of a rod aligned along the *y*-axis. A Kikuchi pattern obtained from this rod is presented in Figure 9b. By using the CrystAlign software, this pattern was assigned to the wutzite phase of ZnO. The same Kikuchi pattern was recorded all along the structure, indicating that crystal orientation was the same, i.e., that they are single-crystal structures. By mapping the Kikuchi patterns on the selected region, indicated by a blue rectangle in Figure 9b, the inverse pole figures (IPFs) for the three X, Y, and Z directions are plotted in Figure 9c. These IPFs show the distribution of the main crystallographic directions that are closer to the X, Y, and Z directions of the reference system. From these results we can see that the growth direction of the rods was mainly along the *c*-axis (<0001> direction). This growth direction is the most typical one observed in wurtzite crystals [1,37]. No difference in the growth direction was observed among the structures grown on the different laser-treated substrates.

**Figure 10:**
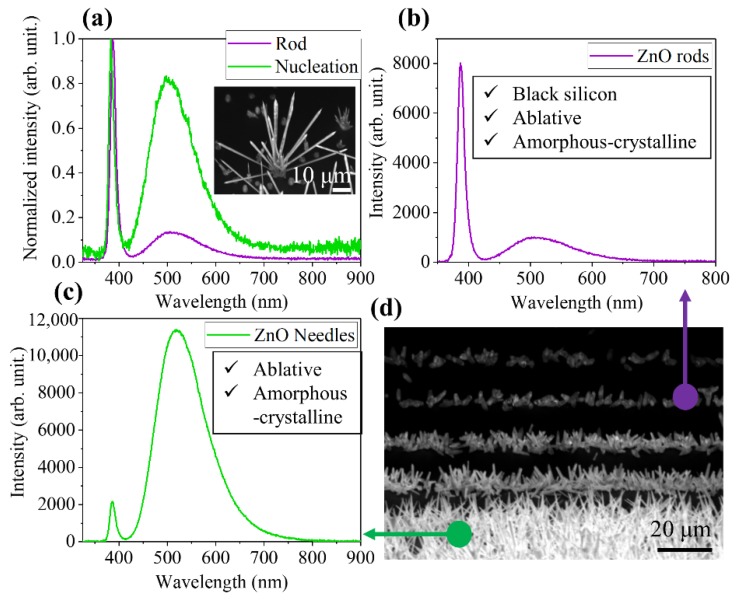
Cathodoluminescence (CL) spectra and images recorded at room temperature on different substrates: (**a**) rods grown on pristine silicon. The inset image shows the CL distribution on the rods. (**b**) Typical CL spectrum recorded on the rod-like structures obtained on the three patterned silicon substrates. (**c**) Typical CL spectrum measured on the needle-like structures grown on ablative and on a–c LIPSS. (**d**) CL image taken on the amorphous-crystalline substrate, showing the distribution of the emission on the structures that grow following the LIPSS scan lines. The sample was tilted 40° to optimize the collection of the signal.

The CL spectra were recorded at room temperature on structures obtained on the different substrates. All the measured spectra had two common emission bands. The first one, centered at 387 nm, was associated with the near band-edge emission (NBE) of ZnO. The second one, with the maximum located at 519 nm, was related to defects, most likely oxygen or zinc vacancies [5,38,39,40]. The CL spectra recorded on the rods grown on pristine silicon showed the dominance of the NBE emission over the defect emission (Figure 10a). Spectra on the nucleation clusters showed an increase in the relative intensity of the defect emission. The higher density of defects in these clusters is very likely related to the accommodation of the difference between the ZnO lattice and the silicon lattice. The CL spectra of the ZnO structures obtained on the patterned substrates showed that the emission was more conditioned by the shape of the structures themselves than by the type of structured substrate used. Rods obtained on the *black silicon*, ablative, and a–c LIPSS had a higher NBE emission intensity in comparison with the defect emission (Figure 10b), whereas needles grown on ablative and a–c LIPSS had a higher relative intensity of the defect emission (Figure 10c). This can be related to the fact that the needles grow closer to the source and the Ar flux inlet (Figure 8) which leads to a higher density of defects in this type of structures, for example, oxygen vacancies. The CL image recorded on the amorphous-crystalline LIPSS substrate (Figure 10d) shows the distribution of the luminescence emission along the lines of the LIPSS with no emission coming for the substrate. In this image, it is also possible to see the evolution of the emission intensity along with the evolution of the structure shapes, being higher the emission from the needles’ (green circle) than from the rods’ (purple circle) morphologies.

## 4. Conclusions

We have shown that it is possible to grow aligned micro/nano-assemblies of ZnO structures on fs-laser-patterned silicon substrates. A comparison was done between the growth produced on patterned substrates combining monocrystalline, amorphous, and polycrystalline silicon. We demonstrated that ZnO micro- and nanostructures preferentially grow on the amorphous Si regions, then on monocrystalline, and finally on polycrystalline silicon. This preferential deposition was associated with a better matching of the ZnO lattice on amorphous silicon along with a slight increase in the local surface roughness of the substrate. The obtained ZnO structures were monocrystalline and showed good crystal quality and luminescent properties that were mostly related to the shape of the structure rather than to the type of silicon substrate employed. These results point toward a simple route for large area ordering semiconductor micro- and nanomaterials, based on the reduction of the effect of lattice mismatch with the substrate by laser-induced local disorder and gentle surface roughness. This strategy can pave the way for growing semiconductor micro/nanostructures on conducting substrates for optoelectronic applications including electroluminescence devices.

## Figures and Tables

**Table 1:** Parameters used to fabricate the different patterned substrates (amorphous-crystalline laser-induced periodic surface structures (a–c LIPSS), ablative low-frequency spatial LIPSS (LSF-LIPSS) and *black silicon*). The pulse energy was measured before the lens.

	Amorphous- Crystalline LIPSS		Ablative LSF-LIPSS	Black Silicon
λ		1030 nm		
Pulse duration		340 fs		
Repetition rate		500 kHz		
Line separation	20 µm		30 µm	3 µm
Scanning speed	2 m/s		0.2 m/s (1 scan)	0.2 m/s (2 scans)
Pulse energy	3.0 μJ		3.4 μJ	3.6 μJ
LIPSS period	1.03 µm		0.84 µm	0.86 µm
